# Effectiveness of Positive Discipline Parenting Program on Parenting Style, and Child Adaptive Behavior

**DOI:** 10.1007/s10578-021-01201-x

**Published:** 2021-07-03

**Authors:** Paul Carroll

**Affiliations:** University of California Merced, 5200 North Lake Rd, Merced, CA 95343 USA

**Keywords:** Positive discipline, Parenting style, Authoritative, Intervention, Effectiveness

## Abstract

In this study, a community sample of parents attending free 7-week Positive Discipline parenting workshops were recruited, as well as a non-randomized community control. Both samples consisted of primarily Hispanic parents with similar demographic information and attrition rates (initial *N* = 91), as well as children of similar age (mean age 6.89 and 6.95 years) and gender. Parenting stress, parenting style, and parent-reported child adaptive behavior were assessed at baseline and after three months. Longitudinal analysis was performed using mixed-effects regression modeling. Results indicate that attendance in Positive Discipline parenting workshops was related to a decrease in authoritarian parenting style, a decrease in permissive parenting style, and a decrease in parental stress. It was also related to an increase in child academic competence, and a decrease in externalizing-hyperactive behavior (both parent-report). These results suggest that positive discipline parenting workshops may alter parenting style and may positively impact children of parents who attend.

## Introduction

The importance of parenting style in child development has been acknowledged for a long time, ever since prototypical concepts were developed to distinguish between certain styles, such as authoritative, authoritarian, and permissive parenting styles [1]. The authoritative parenting style for example, which is the combination of high warmth with high limit-setting, has been linked to children with improved psychosocial maturity, more academic competence, less internalized distress, and less externalizing problems [2] including in samples of serious juvenile offenders [3]. An overly punitive style on the other hand has been linked to increases in both internalizing and externalizing behavior [4], a greater risk of juvenile delinquency [5], and even a greater risk of obesity [6]. Permissive or indulgent parenting style has been similarly linked to a range of undesirable outcomes, from sleep problems in preschool aged children [7], to entitlement, narcissism, and lower work ethic in young adulthood [8]. Yet despite all of this and more, most of the research on parenting style remains correlational. Nor is there very much to demonstrate a method by which it may be modified. If such a method were available though, it might enable a true experimental test of the effects of parenting style, via a direct manipulation, and a follow-up observation.

For now, the strongest evidence of a causal connection between parenting styles and the outcomes they are linked to comes from longitudinal studies that do their best to predict and control for other possible confounds. One such study examined parenting style in parents of preschool aged children, who were followed all the way to early adolescence [9]. After controlling for initial child differences, the study found that certain authoritarian-distinctive power-assertive practices, such as verbal hostility and psychological control, were the most detrimental to children, while the most competent and well-adjusted children tended to have more democratic or authoritative parents [9]. Another longitudinal study of adolescents looked at parenting style and school dropout rates, finding that students with authoritative parents were the most likely to have completed secondary school, and the least likely to have dropped out, after controlling for numerous other variables [10]. This relationship was found to be partly mediated through adolescent’s school engagement, emphasizing the importance of quality parent–child relationships for student’s school engagement [11]. There is therefore reasonable evidence to suggest that a more authoritative and less authoritarian style may be causally related to better outcomes in children—and at least some evidence of the specific mechanisms or mediating factors that drive this relationship. There is less evidence, however, on what interventions may be successful at modifying parenting style, or any of the identified components of it which seem to be harmful or helpful.

Among the very few intervention studies in which parenting style itself was a direct outcome variable and not just a predictor variable, two somewhat similar and yet distinct parenting training programs stand out. One is the widely known, family-centered Triple P program [12, 13], which is possibly the most researched parenting education program in existence [14]. The Triple P program appears to be an effective program at reducing dysfunctional parenting strategies and increasing confidence in parents [12], making it a worthy choice and a promising approach. Another program which is lesser-known but the subject of this study, is the Positive Discipline parenting program [15, 16]. Although much less researched, Positive Discipline is beginning to show similar evidence that it may modify parenting attitudes and behaviors [17, 18]. The first of these studies looked at 101 parents attending Positive Discipline parenting classes and found that parents experienced a decrease in authoritarian and permissive parenting style along with an increase in authoritative style, at 3-months after completion of the program [17] A second study looked at 107 parents of more diverse ethnicity and lower SES and found a similar pattern of effects on parenting style, sustained to three months after attending the workshops [18]. Thus, the Positive Discipline program seems like it may be a promising possibility in modifying parenting behavior—and may even be effective with low SES populations who could possibly benefit from the intervention the most. So, what exactly is Positive Discipline, and how does it differ from the many other existing parenting programs?

Positive Discipline is an approach to raising children that is based on the teachings of Alfred Adler and Rudolph Dreikurs, which emphasizes the need for belonging as a fundamental motivator of human beings [16, 19, 20]. A basic premise of the approach is the understanding that “misbehaving children are discouraged children who have mistaken ideas (faulty private logic) about how to achieve their primary goal—to belong” [21]. Thus, a major focus of Positive Discipline is to help parents and educators understand these mistaken ideas that children may hold, and to use a variety of specific strategies to help children feel a sense of belonging, which is their root goal. For example, there are several main concepts which are emphasized, such as the use of encouragement (not praise), and the use of family and class meetings to solve problems in a democratic manner, which helps children feel a sense of belonging and significance [15]. What makes the Positive Discipline model unique though is the teaching of core concepts through the use of experiential techniques, which help a participant to not only practice a specific skill, but to “feel” what it is like to be on both sides of the approach, versus another approach [22]. The goal of the approach is to achieve effective discipline, which is defined as that which helps children feel a sense of connection, is mutually respectful, is effective long-term, teaches important social skills, and builds a sense of personal capability in children [15, 22].

While the focus of Positive Discipline is mainly to help parents to achieve effective discipline, it is very possible that it may also be helping parents modify their parenting style towards an authoritative style, and away from a permissive or authoritarian style. Many of the specific tools and techniques that Positive Discipline advocates [15, 23] are very similar to or overlap completely with the dimensions of parenting style identified as authoritative by existing parenting dimensions scales, like the Parenting Dimensions Questionnaire [24]. Many items which were empirically derived to characterize authoritativeness in four different sub-dimensions, are directly representative of techniques and virtues extolled by Positive Discipline. For instance, in the warmth/involvement factor, there are items such as “Gives praise when the child is good,” “gives comfort and understanding when the child is upset,” and “expresses affection by hugging, kissing, and holding the child.” These items are analogous to whole sections of techniques recommended in Positive Discipline, like “give encouragement freely,” and “give a hug,” [23]. Each of the other sub-dimensions identified as belonging to authoritative parenting style also have clear analogous representations in Positive Discipline, including Reasoning Induction (“take time for training”), Democratic Participation (“hold weekly family meetings”), and Good Natured / Easy Going (“look for improvement, not perfection”) [23]. Thus, Positive Discipline appears to be very well representative of the authoritative style, at least as it is intended. The question remaining though, is how effective a typical applied workshop is at modifying parent’s behaviors, and if so whether these modifications carry over into benefits in children.

So far, the existing research on Positive Discipline interventions is limited. Jane Nelsen’s 1979 dissertation provided the first trial of what would later form the foundation of Positive Discipline, although this 12-week intervention involved both parents and teachers, and many of the techniques that are taught today had not been developed yet [25]. Other studies have focused on school-based applications of the program, such as implementing class meetings [26], or democratic problem-solving [27]. One study looked at Adlerian-based parenting classes broadly, of which Positive Discipline as a sub-type, but was not focused specifically on Positive Discipline [28]. Only three studies have focused on Positive Discipline parenting trainings in their current and widely delivered format [17, 18, 29], but the results so far are promising. A dissertation by Holliday found that Positive Discipline parenting workshops increased authoritative parenting style (and decreased authoritarian and permissive style), although in relatively affluent participants [17]. A second study in a much lower SES population also found the workshops altered parenting attitudes and behaviors, although using different measures [18]. The effects in either study did not appear to be limited or moderated by common factors such as SES, ethnicity, or being a single parent [18, 29]. Thus, the parenting workshops have the potential to not only improve the long-term prospects of many children, but potentially shrink socio-developmental disparities as well, if applied properly.

The purpose of this study was therefore to replicate and extend previous findings, in a population of mostly low-income parents and community controls, attending free Positive Discipline parenting training workshops (or community controls). One main addition of this study, relative to previous studies, was the recruitment of a non-randomized control group—making it a two group quasi-experimental design. This type of control cannot eliminate personal confounds, but it can protect against some threats to internal validity such as testing effects, maturation, and history threats. Another main addition was the use of new and extensive measures, including both extensive measures on parenting style and parent-report of their children’s adaptive behavior, broken into several domains. Theoretically, several of these domains correspond directly to parenting style, such as academic competence, and types of both internalizing and externalizing behavior [2]. The study collected mail-in questionnaires at baseline, and after three months.

The primary hypotheses of this study were that authoritative parenting style would increase in the intervention group, but not in the control, while authoritarian style, permissive style, and parenting stress would decrease in the intervention group but not in the control. These findings would serve to replicate and extend previous results. Secondary hypotheses concerned outcomes in children—where it was hypothesized that academic and social competence would increase in the intervention group but not in the control, while internalizing and externalizing behavior would decrease in the intervention group, but not the control. These hypotheses are theoretically implicated but have not been examined. Beyond that, exploratory analyses would examine the many sub-domains within each dimension of parenting style, as well as the sub-domains within each major dimension of children’s adaptive behavior—without any specific hypotheses of effects. Of course, a major limitation that must be understood is that all the data are parent reported, as well as the comparison group being a non-randomized comparison. Nonetheless, the measurement of these outcomes is a step forward in connecting theoretical desirable endpoints, and a two group quasi-experiment is still an improvement over a one-group quasi-experiment. This study therefore serves the aim of a program of research, which is to seek to successfully modify parenting style to reduce negative influences on children, to observe the positive effects of this change in parenting style on children, and to understand and optimize the maximum benefit possible to render via this approach.

## Method

### Participants and Procedure

In this study, parents invited to participate in a free, seven-week workshop on Positive Discipline were recruited by a community partner (a local non-profit service provider), while a comparison group of parents involved in local adult schools where recruited directly by the research team. The comparison group in this study was therefore a non-randomized control group, but both samples consisted of primarily Hispanic, Spanish speaking parents, and had similar demographic information, attrition rates, and children of similar age and gender. Participants where recruited from a total of 18 sites, in two waves. Outreach efforts for the workshops varied by site, but consisted of contact with community leaders, distributed flyers, direct outreach by facilitators, and through word of mouth. Outreach efforts for the non-randomized control group consisted of showing up with permission to classes being taught at the adult school, introducing the study and asking if there were any parents of young children who would be interested in participating, and soliciting names and addresses of those interested.

**Fig. 1:**
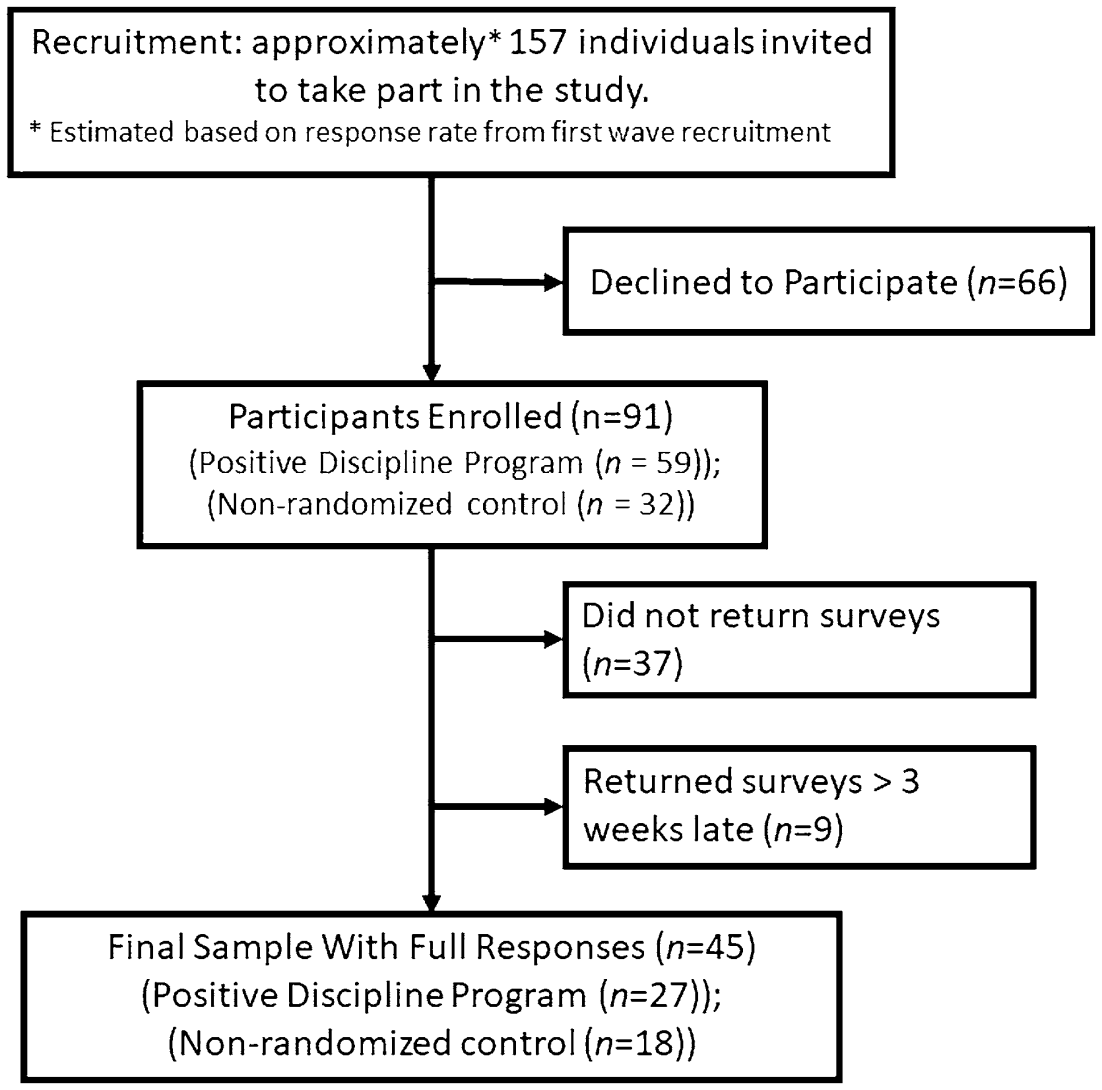
Recruitment of study participants

In the first wave of recruitment, after the names and addresses of those interested were gathered in person, potential participants were mailed an initial packet with more information about the study, a consent form, and an initial survey. Fifty-two participants chose to return the initial survey and were enrolled in this manner, which was a response rate of approximately 58 percent. In the second wave, interested parties were simply given a self-addressed stamped envelope with the initial packet of information, consent form, and survey—and enrolled if they chose to fill it out and return it. An additional thirty-nine participants were enrolled in this manner, for an initial total of *N* = 91 participants. Three months after initial contact, a follow-up survey was mailed to the address on file for each participant, and a total of 54 were returned (37 were not returned), which is an approximately 59% retention rate. However, participants who returned surveys more than three weeks late at either timepoint (*n* = 9) were further restricted from the analysis of primary outcomes, although their demographics are reported. Thus, the initial sample was 91 participants, the final sample after attrition was 45 total participants—while the useable sample which included useable primary outcome data from either timepoint, was 82 participants. A flowchart of the recruitment process is shown in Fig. 1.

A mail-in survey was chosen as the primary data collection tool in this study for two reasons. One—it limited the potential for facilitators of parenting workshops to bias the results, as all responses would be filled out at home. And two—it made sure there was adequate time to complete each survey, which were each 8 pages in length, plus a tear-off page. To ensure confidentiality and to limit potential experimenter bias, participants did not put their name or address anywhere on the bulk of the survey, but instead only wrote that information on a final tear-off page of each survey—which asked where they should have a gift card mailed to for their participation. The surveys were then self-addressed back to a neutral third party, who physically separated names and addresses from each survey, writing a codename on each one and delivering them in batches to the principal investigator. Thus, anonymity of the participants was maintained while allowing responses to be matched by their codename, and any experimenter bias in the coding or judgement of survey responses should have been limited. Each participant also read and signed a consent form, which was also separated from their responses and stored separately. For their participation, each participant was mailed a generic $10 gift card for each response, for a total of $20 in incentives if they completed both surveys.

The overall design of the study was thus a pre-test, post-test design, with a non-randomized control. Most of both samples were Hispanic, and approximately half of those recruited were Spanish speaking. Because the parenting workshops were being offered primarily as a community service, not as a research project, it was not ethical or feasible to randomize any participants to not receive the workshops—but a semi-equivalent comparison group was nonetheless recruited. While not as ideal as a randomized control, the non-randomized control could still act as a control against several possible threats to validity, including a simple testing effect, maturation in the sample, or natural attrition causing those with deteriorating life circumstances to drop-out of the study. Since it was known from previous research that attrition was likely to be very high in this very low SES sample [18], it was hoped that the non-randomized control would experience equivalent rates of attrition, and thus somewhat protect against this threat. Similarly, if a simple testing effect or maturation over the three-month follow-up had any effect, then the non-randomized control group should experience this effect as well. And indeed, as can be seen in the results, both groups were similar in demographic characteristics, had similarly aged children, and had similar rates of attrition—hopefully mitigated some of these threats to validity.

### Measures

The survey measures included several demographic variables including age, gender, ethnicity, education, income, financial strain, number of children, and single parent status. Positive Discipline specific attitudes and behaviors were assessed using the 7-item Positive Discipline Parenting Scale (PDPS), which has shown acceptable internal consistency for community research (α = 0.775–0.772) [30]. Parental stress and reward were assessed using the 12-item Spanish adaptation of the Parental Stress Scale (PSS), which has also shown acceptable internal consistency (α = 0.76–0.77) [31], which breaks down into the sub-domains of stress and reward from parenting. The main dimensions of of authoritative, authoritarian, and permissive parenting style were assessed using the 62-item Parenting Dimensions Questionnaire-Revised (PDQ-R) [24], which also breaks those main dimensions down into 11 sub domains. The sub-domains for authoritative parenting style include “warmth/involvement,” “reasoning induction,” “democratic participation,” and “good natured/easygoing.” The sub-domains for authoritarian parenting style include “verbal hostility,” “corporal punishment,” “non-reasoning/punitive strategies,” and “directiveness.” The sub-domains for permissive parenting style include “lack of follow through,” “ignoring misbehavior,” and “self-confidence.” (reverse coded) [24]. The PDQ-R has been shown to have acceptable to excellent internal consistency for the main parenting style dimensions (α = 0.75–α = 0.91), while the sub-dimensions are more exploratory. These measures are the sum of what we asked parents to describe about themselves, and their parenting style.

Next, we asked each parent to describe their youngest two children who were at least 3–18 years old, using a 54-item short form of the Child Adaptive Behavior Inventory (CABI) [32]. The short form used measures 20 individual attributes, such as task orientation, creativity, introversion, extroversion, so on—which are then z-scored and combined into six main factors of adaptive behavior. These six main factors of adaptive behavior assess (1) Academic competence; (2) Social competence; (3) Externalizing-aggressive; (4) Externalizing-hyperactive; (5) Internalizing—socially isolated; and (6) Internalizing—psychological symptoms (anxiety, depression, etc.). The CABI has been validated in samples of children from age 4 in preschool to age 12 in 4^th^ grade, with samples of up to *n* = 1723 [32, 33]. In this context, the six main domains are considered reliable for research purposes, while the 20 smaller domains are somewhat less reliable, and considered only for exploratory purposes. The only other information we asked parents to provide were the age and gender of each child.

### Treatment

Each seven-week workshop was carried out by a pair of trained facilitators, all of whom had been trained and certified by the Positive Discipline Association as Certified Positive Discipline Parenting Educators (a fourteen-hour certification) and had at least some previous experience facilitating workshops. All facilitators at Spanish-language sites were bilingual and had access to Spanish language materials. In addition, these facilitators were generally recruited and trained from the same cultural background as the program participants, and therefore had an enhanced cultural competency to be able to relate to and help program participants with their unique challenges. The structure of each workshop is flexible by design, but generally includes warm-up activities, lessons, experiential activities, and parents-helping-parents in every 2-h session. Sessions are typically focused on hands-on experiential learning, often with the use of props and visual aids, but without any standardized audio-visual presentation, such as DVDs or recorded lectures, in this format. Many of the activities involve participants role-playing both as children and adults, as they process different parenting behaviors and their reactions to them from both perspectives [34]. Facilitators tailor each session to the needs and challenges of each group however, with different warm-ups, lessons, and more than 70 possible experiential activities in a facilitators training manual to choose from [34]. The goal is to help parents overcome the particular challenges they are facing to effective discipline, while becoming both kind and firm. To ensure confidentiality and allow the process to take place with potentially sensitive disciplinary issues, members of the research team did not attend the sessions beyond the initial recruitment visits. Those seeking additional information about a typical parenting workshop of this kind should consider reaching out to the Positive Discipline Association [22], or individual workshop facilitators.

### Data Analysis

The primary hypotheses of this study were that authoritative parenting style and positive discipline parenting style would increase in the intervention group, but not in the control, while authoritarian style, permissive style, and parenting stress would decrease in the intervention group but not in the control. A mixed effects regression model was used for each of these outcomes, with subject as a random effect and both group and group by time interaction as fixed effects, using an unstructured covariance matrix. A mixed effects model accounts for the intra-class correlation between repeated measures by subject, allows for them to vary as a random effect, and controls for the imbalance of observations as a result of attrition—without restricting the sample to only participants with complete data [35]. This preserves statistical power as opposed to dropping all the observations that did not complete both time points. Including a fixed effect for group models the overall differences between comparison groups which exist at the outset and is appropriate for a non-randomized control. The group by time interaction is thus the primary outcome of importance—which estimates if the treatment group experienced any significant changes over time, and if they varied significantly from changes experienced by the comparison group. This significance of this interaction was assessed using a one-tailed *F*-test, since there was a directional hypothesis regarding the interaction.

The secondary hypotheses of this study were that academic competence and social competence would increase in children of parents in the intervention group, but not the control, while the domains of externalizing (aggressive), externalizing (hyperactive), internalizing (socially isolated), and internalizing (psychological symptoms) would decrease in the children of parents in the intervention group, but not the control. A mixed-effects regression model was also used for these outcomes, with the same group and group by time interaction modeled as fixed effects. Finally, several exploratory analyses were then conducted using the same mixed-effects regression model, but for each of the 11 sub-domains of the PDQ-R, and each of the 20 sub-scales of the CABI. Since these subscales are likely to be of lesser reliability and since they were not the primary outcomes of the study, the results of these analyses should be considered exploratory—and the significance of any findings should be considered preliminary, given the large number of significance tests. These analyses are nevertheless included though, to aid in a more theoretical understanding of the results, and to guide future directions of research.

## Results

### Demographics

Demographic information for both groups at time one is reported in Table 1. Of the 91 participants originally recruited, approximately 81% were female, 75% were Hispanic, and 18% reported being a single parent. Highest level of education achieved was about average for the region, with 26% reporting that they had not completed high school, 74% having completed high school or greater, and 16% having obtained a four-year degree or higher. Income was also very low on average, with 74% reporting a combined household income of less than $39,999/year (near the median for the region), and 47% reported that they “sometimes struggle with finances” or that “financial worries are a serious problem” for them each month. The number of children participants had ranged from 1 to 8, with a mean of 2.51. The average age of participants was 35.9. The only significant demographic difference between groups was that there were more women in the treatment versus the comparison group; *t* (79.4) = 2.67, *p* = 0.009. Otherwise the groups were fairly similar demographically, although group differences were expected and statistically controlled for as a fixed effect regardless.

**Table 1:** Demographic characteristics of participants

Demographic variable	Treatment group (*n* = 59*)*	Comparison (*n* = 32)
Age (*M, SD*)	36.4 (11.25)	35.1 (7.99)
Number of children (*M, SD*)	2.34 (1.36)	2.81 (1.18)
Hispanic (%)	44 (75)	24 (75)
Female (%)	44 (75)	30 (94)
Education: high school or greater (%)	45 (78)	21 (68)
Education: 4-year degree or higher (%)	6 (10)	8 (28)
Income: < $39,999	47 (81)	20 (63)
Financial worry (%)	30 (52)	13 (42)
Single parent (%)	11 (19)	5 (16)

Individual variables may have missing data, and lower individual *n*’s

### Primary Outcomes

The primary hypotheses of this study were that authoritative parenting style and positive discipline parenting style (PDPS) would increase in the intervention group but not in the control, while authoritarian style, permissive style, and parenting stress would decrease in the intervention group but not in the control. These hypotheses were tested using a mixed-effects regression model, and the results and parameter estimates for each variable are displayed in Table 2. The analysis revealed a significant group by time interaction for PDPS; *F* (2, 50.45) = 4.01, *p* = 0.024, for authoritarian style; *F* (2, 53.45) = 8.74, *p* = 0.001, for permissive style; *F* (2, 49.34) = 7.09, *p* = 0.002, and for parental stress; *F* (2, 46.62) = 3.10, *p* = 0.054,^1^ but not for authoritative style—which was estimated to increase, but not significantly; *F* (2, 49.89) = 0.63, *p* = 0.535. These variables were transformed into z-scores, and z-scored parameter estimates for the treatment group are provided in Table 2. None of these outcomes changed significantly for the comparison group, indicating that the changes do not appear to result from a simple testing effect, maturation, or other methodological artifact that would equally influence the comparison condition.

**Table 2:** Results for main outcomes in parents

Parameter	Estimate	95% CI (Lower/Upper)	Sig	Interaction Sig
PDPS*	.408	0.095/0.717	.011*	.024*
Authoritative style	.154	− 0.125/0.433	.274	.535
Authoritarian style*	− .493	− 0.729/ − 0.256	.000*	.001*
Permissive style*	− .514	− 0.789/ − 0.239	.000*	.002*
Parental stress*	− .284	− 0.515/ − 0.053	.017*	.054^†^

^*^Significant at *p* < .05, ^†^Significant at *p* < .05 given a directional hypothesis

Next, the second set of hypotheses were that academic competence and social competence would increase in children in the intervention group but not the control, while the domains of externalizing (aggressive), externalizing (hyperactive), internalizing (socially isolated), and internalizing (psychological symptoms) would decrease in the children of parents in the intervention group but not the control. These hypotheses were also tested using a mixed-effects regression model, and the results and parameter estimates for each variable are displayed in Table 3. The analysis revealed a significant group by time interaction for academic competence; *F* (2, 77.81) = 5.36, *p* = 0.007, and for externalizing (hyperactive); *F* (2, 74.86) = 3.54, *p* = 0.034, but not for any of the other four domains of adaptive behavior. Transformed (z-scored) parameter estimates for the children in the treatment group are provided in Table 3. As expected, none of these outcomes changed significantly for the comparison group, indicating that constructs in question appear stable, lacking an intervention.

**Table 3:** Results for main outcomes in children

Parameter	Estimate	95% CI (Lower/Upper)	Sig	Interaction Sig
Academic competence*	.432	0.158/0.705	.002*	.007*
Social competence	.225	− 0.059/0.509	.119	.194
Externalizing—aggressive	− .165	− 0.387/0.057	.144	.320
Externalizing—hyperactive*	− .288	− 0.513/ − 0.063	.013*	.034*
Internalizing—socially isolated	− .193	− 0.422/0.036	.098^†^	.221
Internalizing—psychological symptoms)	− .094	− .360/0.172	.482	.363

^*^Significant at *p* < .05, ^†^Significant at *p* < .05 given a directional hypothesis

### Exploratory Analysis

Finally, several exploratory analyses were then conducted using the same mixed-effects regression model, but for each of the 11 sub-domains of the PDQ-R (Table 4), and each of the 20 sub-scales of the CABI (Table 5). These results presented with z-scored parameter estimates for ease of interpretability and comparison. On the parenting dimensions questionnaire, the largest single effect was a large increase in parenting self-confidence (reversed as “low self-confidence”, part of the Permissive Style), while there were several other decreases including in verbal hostility, corporal punishment, directiveness, and the use of non-reasoning and punitive strategies (all part of the Authoritarian Style). All observed changes in the treatment group were in the hypothesized and positive direction, and there were no significant changes in the comparison group, despite the increased odds of type I error. On the child’s adaptive behavior questionnaire, there were several notable changes of roughly one quarter to a half a standard deviation in size, including reported decreases in distractable and antisocial behavior, and increases in kindness, intelligence, and creativity (all parent-report), as well as possible decreases in social isolation and increases in social perception. These results should be considered preliminary pending a replication—but it seems unlikely that several of them are due to error. There were no significant changes in the children in the comparison group—despite the numerous analyses and the increased odds of type I error. This would seem to suggest that spontaneous changes in these traits are rare outside of some type of specific influence, such as parenting class.

**Table 4:** Results for exploratory outcomes in parents

Parameter	Estimate	95% CI (Lower/Upper)	Sig	Interaction sig
Warmth and involvement	.139	− .161/.440	.356	.648
Reasoning/induction	.006	− .287/.298	.970	.799
Democratic participation	.218	− .089/.525	.160	.347
Good natured/easy going^†^	.256	− .028/.540	.077^†^	.154
Verbal hostility^†^	− .284	− .568/.001	.051^†^	.139
Corporal punishment*	− .427	− .674/ − .179	.001*	.004*
Non-reasoning/punitive*	− .387	− .640/ − .135	.003*	.012*
Directiveness*	− .396	− .720/ − .073	.017*	.053^†^
Lack of follow-through^†^	− .255	− .520/.010	.059^†^	.164
Ignoring misbehavior	− .208	− .550/.134	.229	.478
Low self confidence*	− .661	− .962/ − .359	.000*	.000*

^*^ Significant at *p* < .05, ^†^Significant at *p* < .05 given a directional hypothesis

**Table 5:** Results for exploratory outcomes in children

Parameter	Estimate	95% CI (Lower/Upper)	Sig	Interaction Sig
Fair	.126	− .188/.440	.427	.651
Kind*	.428	.127/.728	.006*	.018*
Anxious	− .219	− .505/.068	.132	.319
Hyperactive	− .127	− .366/.112	.294	.554
Antisocial*	− .297	− .530/ − .064	.013*	.034*
Oppositional	− .200	− .477/.077	.154	.343
Hostile	.018	− .189/.226	.859	.926
Intelligent*	.432	.171/.692	.001*	.004*
Creative*	.436	.149/.723	.003*	.007*
Task oriented	.232	− .080/.544	.142	.291
Distractable*	− .376	− .631/ − .121	.004*	.011*
Extroverted	.140	− .105/.386	.257	.523
Introverted	− .110	− .356/.156	.374	.671
Depressed	.121	− .107/.348	.294	.351
Somaticized	− .157	− .449/.135	.289	.307
Impaired development	.067	− .161/.295	.559	.825
Socially isolated^†^	− .296	− .591/ − .001	.049*	.092^†^
Socially rejected	.032	− .298/.362	.845	.854
Socially perceptive^†^	.314	.021/.607	.036*	.098^†^
Socially skilled	− .100	− .409/.209	.522	.590

^*^Significant at *p* < .05, ^†^Significant at *p* < .05 given a directional hypothesis

## Discussion

Taken together, the findings from this study indicate that parenting style did indeed change significantly over time in conjunction with Positive Discipline parenting workshops, including decreases authoritarianism and permissiveness—as well as decreases in parental stress, and increases in positive discipline style parenting. The changes do not appear to be a result of a possible testing effect, maturation, or other factors that would equally influence the comparison condition. Furthermore, the findings appear to indicate a reduction in externalizing—hyperactive behavior, and an increase in academic competence, in children of parents attending the Positive Discipline workshops. While some other hypotheses were not supported, overall these are promising results that may be of interest to both parents and educators.

These findings are consistent with previous research in their demonstration of an effect on parents [17, 18], but also extend those findings with the measurement of outcomes in children, and the use of a control group. The finding of increased academic competence and decreased hyperactive behavior are consistent with research that shows authoritative parenting style (and lower permissive or authoritarian style) predicts school readiness and achievement in the first grade [36]. Although there was only a three-month follow up in this study, parenting style has also been shown to predict dropout rates in secondary school [10, 11], making these early childhood interventions of potentially far-reaching importance. More broadly speaking, this research adds to the base of support for implementing effective parenting programs in general, [12, 13]. Hopefully future research will continue to explore such programs with experimental studies, and extended follow-up observations.

Of course, the present study has some limitations. First, it used a nonrandomized control group rather than a randomized control, which introduces the possibility of selection bias into the intervention group—and cannot rule out the possibility of personal confounds contributing to the observed effects. It is possible that parents attending the parenting workshops were simply motivated to improve, and this may be responsible for some or even all their improvement. However, the lack of *any* significant changes in any of the observed variables in the control group suggests that these traits are relatively stable, lacking a directed intervention. Another limitation was the use of entirely parent-reported measurements. Although the study used validated measures for all reported outcomes, they are nonetheless self-reported, and may be influenced by participant response bias. At the very least, the comparison group somewhat protects against this threat—as the parents in this condition might also be motivated to report socially desirable information about themselves or their children, and yet reported no changes. Another limitation was the relatively low prevalence of single-parents taking the workshops. It is not known how well these results would generalize to other demographic groups, such as single parents—although previous research has not found outcomes to be moderated by single-parent status [18, 29]. Some final notable limitations include the relatively small sample, high attrition, and a relatively short 3-month follow-up period. All of these limitations represent the challenges of doing field research with vulnerable, low-resource participants—and yet future research should attempt to do better in these areas if possible.

Despite the limitations, this study demonstrated several significant changes in parenting style for the participants who attended Positive Discipline parenting workshops—which is not something that many programs have been able to accomplish. It also appeared to show sizeable increases in children’s academic competence, as well as potential improvements in several types of adaptive behavior, which are all desirable outcomes. It is possible that there are unique features to Positive Discipline that help parents make a philosophical switch, such as the central concept that a misbehaving child is a discouraged child, and that their behavior will improve when they feel their need for belonging being met [15]. Or perhaps it is possible that it is the *way* that these concepts are taught, through activities like role-playing and experiential exercises, which have been invented and refined over the years by dozens of individual facilitators [34]. Either way, there appears to be a high level of consumer satisfaction with parents who have attended a Positive Discipline workshop, and a relatively low cost per participant to implement [18]. Hopefully future research can continue to explore this cost versus benefit, as well as the longer-term effectiveness, if any, of conducting Positive Discipline training workshops.

A few of the questions that remain to investigated include whether there is an optimum dose of the training, whether the effects are sustained, and whether there is any change in Authoritative parenting style specifically, which has seen inconsistent results in two different studies. It may be that the average effect on Authoritativeness lies somewhere between what was observed in this study and one previously [17], or it’s possible that the greatest part of the effect is delayed, as was observed previously. Perhaps future research could explore if there is an optimum number of Positive Discipline workshops to expose participants to before they reach a point of diminishing interest or benefits, or if there is an optimum number of participants to have in each group setting? Information like this would greatly aid stakeholders who might wish to offer these classes to the population they serve, such as school-districts or human services agencies. The greatest need for future research is probably still for a well-conducted randomized controlled trial, with a longer-term follow-up, and multiple third-party observations. Such as study would ultimately offer the strongest validity to its conclusions and produce the best estimates of the program’s effectiveness.

There may ultimately be several different parenting programs that are effective, and each may be effective in different ways or with different populations. Hopefully this research contributes to the overall goal of finding out what programs exist that are effective at all—and isn’t seen as placing Positive Discipline in competition with any other particular program. Indeed, as some researchers have suggested, it is time to start treating investments in parenting style as investments in human development [37] and when making such investments there is always room for different approaches.

## Summary

Positive Discipline is a type of parenting program based on Adlerian psychology, which emphasizes a style of parenting that is analogous to authoritative parenting, though somewhat unique. This study is the first to examine the effects of Positive Discipline parenting workshops on outcomes in children of those attending, as well as the first to incorporate a control group (albeit non-randomized). The results indicate that attendance in Positive Discipline parenting workshops was related to a decrease in authoritarian parenting style, including all four subcomponents (verbal hostility, corporal punishment, non-reasoning punitive, and directiveness), as well as a decrease in permissive style parenting, and a decrease in parental stress. It was also related to an increase in child academic competence (parent-report), and a decrease in externalizing-hyperactive behavior (parent-report). These results suggest that Positive Discipline parenting workshops appear to be effective at improving parenting style in the studied population and may further positively impact the children of parents who attend. It should continue to be implemented and explored.

## Footnotes

1 P-values reflect two-tailed significance test. Result is significant at *p* < .05 in light of a directional hypothesis.
